# Acute fatty liver of pregnancy with transient resolution preceding postpartum liver failure requiring liver transplant

**DOI:** 10.1515/crpm-2024-0028

**Published:** 2024-10-08

**Authors:** Rachel Lee, Kimberly Herrera, James Bernasko

**Affiliations:** Department of Maternal Fetal Medicine, Stony Brook University Hospital, Stony Brook, NY, USA

**Keywords:** acute fatty liver of pregnancy, fulminant hepatic failure, orthotopic liver transplant, intrahepatic cholestasis of pregnancy, atypical pre-eclampsia, hepatic encephalopathy

## Abstract

**Objectives:**

Acute fatty liver of pregnancy (AFLP) is a rare, potentially fatal complication of unknown etiology that occurs in the third trimester or early postpartum and can be associated with adverse maternal and fetal outcomes. The purpose of this report is to share a case of AFLP in which a period of objective and symptomatic resolution preceded delayed postpartum liver failure and liver transplant.

**Case presentation:**

A 35-year-old G3P0020 female experienced preterm premature rupture of membranes (PPROM) at 32 weeks’ gestation and AFLP. She delivered vaginally and despite apparent initial disease resolution, was found 22 days later to have fulminant acute liver failure that required liver transplantation.

**Conclusions:**

AFLP should be monitored closely postpartum even if disease parameters initially appear to resolve after delivery.

## Introduction

Acute fatty liver of pregnancy (AFLP) is typically most severe early in the disease course and resolves rapidly if delivery is prompt [1]. No cases have been reported in the English literature in which clinical condition and serum transaminases improved significantly for several days postpartum before acute exacerbation and fulminant hepatic failure required orthotopic liver transplant.

## Case presentation

A 35-year-old G3P0020 female was admitted at 32 weeks’ gestation following spontaneous rupture of membranes. She endorsed generalized pruritus, fatigue, and decreased appetite for two days, but denied abdominal pain, fever, chills, emesis, diarrhea, recent travel or sick contacts. She reported two prior surgical terminations of pregnancy. Physical examination was unremarkable except for scleral icterus and dark urine. Her vital signs on admission were: temperature 36.9 °C, heart rate 93 bpm, 17 respirations/minute, blood pressure 130/86 mmHg, and 100 % O_2_ saturation on room air. Fetal assessment revealed a reactive tracing with 145 bpm and moderate variability. Urine protein was negative, but alanine transaminase (ALT) and aspartate aminotransferase (AST) were significantly elevated to 1,808 and 2,012 respectively and bile acids were 409. Betamethasone was administered to accelerate fetal lung maturity. The differential diagnosis included AFLP, intrahepatic cholestasis of pregnancy (ICP) and atypical pre-eclampsia. ALT and AST were 1,741 and 2,010 respectively 3 h after admission. Correspondingly, glucose was 87 mg/dL, ammonia was 32 umol/L, and fibrinogen was 471 mg/dL. Hepatitis A, B, C viral serology, autoimmune hepatitis, alpha-1 antitrypsin, hereditary hemochromatosis, ceruloplasmin, acetaminophen, ethanol, urine toxicology, thyroid function tests, creatinine kinase, uric acid, tissue transglutaminase antibodies IgA and IgG were normal. Urinalysis confirmed bilirubin and trace protein. Abdominal ultrasound revealed non-dilated bile ducts (indicating no biliary obstruction) and no sonographic evidence of fatty liver. Liver biopsy was not recommended. The differential diagnosis was narrowed to AFLP vs. ICP, and Ursodiol 300 mg TID was started.

Labor ensued spontaneously on HD #2 resulting in vaginal delivery of a viable male fetus with Apgar scores of 5 and 7 at 1 and 5 min, respectively. Ampicillin, gentamicin, and clindamycin were started during labor for suspected intra-amniotic infection and continued for 24 h postpartum. Placental pathology was consistent with acute chorioamnionitis. On postpartum day 2, there was no evidence of encephalopathy, ALT and AST had decreased to 511 and 211 respectively, total bilirubin was 11.7, and INR was 1.3 (Table 1). Patient was asymptomatic and scleral icterus had improved; therefore, Ursodiol was discontinued and patient was discharged home on HD #5 with outpatient gastroenterology and hepatology follow-up.

**Table 1:** Laboratory values during the disease course.

	First admission (pre-delivery)	First discharge (post-delivery)	Second admission	Normal range
Platelets, k/µL	236	274	258	150–350
AST, IU/L	2,012	211	1,032	0–32
ALT, IU/L	1,808	511	893	0–33
Total bilirubin, mg/dL	16.1	11.7	25.3	0–1.2
Alkaline phosphatase, IU/L	275	213	230	39–117
Bile acids, µmol/L	409	–	–	0–10
INR	1.8	1.3	5.8	0.8–1.2
Glucose, mg/dL	87	100	58	70–99

Patient was recovering well until postpartum day 22 when she experienced sudden-onset low grade fever, chest pain, nausea, vomiting, fatigue, and loss of appetite, prompting her to present to the emergency room. Scleral icterus had deepened, ALT and AST were 893 and 1,032 respectively, total bilirubin was 23.4, and INR was 6.5. Encephalopathy was rapidly worsening despite normal imaging of the liver; therefore, emergency liver transplant was performed.

Patient underwent uncomplicated deceased donor liver transplant. Liver pathology report returned as extensive hepatocyte necrosis consistent with the clinical impression of AFLP. Postoperative course was uncomplicated, and patient was discharged home after normalization of LFTs.

## Discussion

Early diagnosis and effective treatment of AFLP is crucial for improving maternal and fetal/neonatal outcome 1], [2. Mortality has improved dramatically since 1965, with the regression of symptoms and normalization of laboratory values, resulting in “reversible peripartum liver failure” [3]. Although AFLP can spontaneously resolve, it continues to be a potentially fatal complication with symptoms starting 1–2 weeks before hospitalization 4], [5], [6 and has been associated with fetal demise [7]. Although AFLP is reported in only 1 of 7,000 pregnancies [7], maternal mortality remains as high as 7–18 % 8], [9; therefore, it is paramount to consider AFLP in the differential of antenatal significant liver function test (LFT) elevations.

One case report recognizing the overlap between AFLP and ICP used liver biopsy to guide management [10]. History, physical exam, and relevant lab/imaging results are usually sufficient to diagnose AFLP and liver biopsy is rarely required [4] given its invasive nature.

The authors of one cohort study of twenty-five patients concluded that specific long-term follow-up after recovery from AFLP is not required if LFTs normalized and patients remained well [11]. Our patient, despite LFTs and symptoms improving within 2 days after delivery, developed liver failure on postpartum day 22. This case of AFLP appeared to resolve after delivery but then acutely worsened and required liver transplant, highlighting the importance of recognizing delayed postpartum complications of AFLP and supporting continued close surveillance even if symptoms and laboratory values improve initially.

### Take-home messages

1) AFLP can appear to improve for weeks following delivery before suddenly worsening, contrary to common impression that the worst manifestation occurs early in the disease course.
2) AFLP management ideally requires early diagnosis, treatment, and hepatology consultation to consider liver transplantation, if necessary.
